# CLRM-10. THE INTER-RELATIONSHIP BETWEEN MULTI-MODAL LONGITUDINAL BIOMARKERS OF NEURAL DAMAGE, INFLAMMATION, AND COGNITION AFTER CAR T CELLULAR THERAPY: A SINGLE-CENTER PROSPECTIVE OBSERVATIONAL TRIAL (NCT04614987)

**DOI:** 10.1093/noajnl/vdab112.009

**Published:** 2021-09-21

**Authors:** Omar Butt, Alice Zhou, Ken Lee, Gregory Wu, Sheng-Kwei Song, Jian Campian, John DiPersio, Beau Ances, Armin Ghobadi

**Affiliations:** Washington University, Saint Louis, USA

## Abstract

**BACKGROUND:**

Immune effector cell associated neurotoxicity syndrome (ICANS) remains a devastating, frequent complication of chimeric antigen receptor (CAR) T cell therapy for advanced-stage hematologic malignancies. Symptoms range from encephalopathy and headaches to aphasia, strokes, and diffuse cerebral edema. Persistent mild cognitive symptoms have also been reported. Unfortunately, the underlying pathophysiology driving ICANS is poorly understood. Current proposed models center on systemic inflammatory changes leading to endothelial dysfunction, blood-brain barrier (BBB) breakdown, and systemic cytokine and/or monocytes infiltration into the central nervous system (CNS). However, these models do not integrate predisposing risk factors for the development of ICANS. We previously demonstrated that pre-infusion plasma neurofilament light chain (NfL), a marker of neurodegeneration, may predict development of ICANS. Early elevations in NfL suggest development of ICANS is also related to pre-existing neuroaxonal injury. The longitudinal relationship between latent neuroaxonal injury, blood brain barrier (BBB) integrity, neuroinflammation, and cognition remains unknown.

**METHODS:**

This prospective, observational trial examines the relationship between multi-modal (blood, cerebrospinal fluid (CSF), neuroimaging) biomarkers and cognition in a cohort of twenty patients undergoing standard-of-care CAR T cellular therapy. Biomarkers for neural injury include blood and CSF NfL and volumetric measures derived from structural magnetic resonance imaging (MRI). Biomarkers for neuroinflammation include blood and CSF glial fibrillary acidic protein (GFAP) and qualification of white matter hyper-intensity burden on MRI. BBB integrity will be quantified using the serum/CSF albumin ratio. Finally, neuropsychological performance testing will assay cognitive performance across multiple cortical domains including attention, memory, and executive function. Participants will undergo a baseline (pre-infusion) examination, followed by evaluation (blood draw, voluntary lumbar puncture, MRI scan, and cognitive testing) on post-infusion day 3 (D3), D30, D90, and D180. The primary outcome is percent change in a given biomarker level.

**RESULTS/CONCLUSIONS:**

This ongoing trial has 2 of 20 planned participants enrolled.

